# AL Amyloid Imaging and Therapy with a Monoclonal Antibody to a Cryptic Epitope on Amyloid Fibrils

**DOI:** 10.1371/journal.pone.0052686

**Published:** 2012-12-26

**Authors:** Jonathan S. Wall, Stephen J. Kennel, Angela Williams, Tina Richey, Alan Stuckey, Ying Huang, Sallie Macy, Robert Donnell, Robin Barbour, Peter Seubert, Dale Schenk

**Affiliations:** 1 Department of Medicine, University of Tennessee Graduate School of Medicine, Knoxville, Tennessee, United States of America; 2 Department of Radiology, University of Tennessee Graduate School of Medicine, Knoxville, Tennessee, United States of America; 3 Department of Biomedical and Diagnostic Sciences, University of Tennessee College of Veterinary Medicine, Knoxville, Tennessee, United States of America; 4 Neotope Biosciences, South San Francisco, California, United States of America; Uppsala University, Sweden

## Abstract

The monoclonal antibody 2A4 binds an epitope derived from a cleavage site of serum amyloid protein A (sAA) containing a -Glu-Asp- amino acid pairing. In addition to its reactivity with sAA amyloid deposits, the antibody was also found to bind amyloid fibrils composed of immunoglobulin light chains. The antibody binds to synthetic fibrils and human light chain (AL) amyloid extracts with high affinity even in the presence of soluble light chain proteins. Immunohistochemistry with biotinylated 2A4 demonstrated positive reaction with ALκ and ALλ human amyloid deposits in various organs. Surface plasmon resonance analyses using synthetic AL fibrils as a substrate revealed that 2A4 bound with a K_D_ of ∼10 nM. Binding was inhibited in the presence of the –Glu-Asp- containing immunogen peptide. Radiolabeled 2A4 specifically localized with human AL amyloid extracts implanted in mice (amyloidomas) as evidenced by single photon emission (SPECT) imaging. Furthermore, co-localization of the radiolabeled mAb with amyloid was shown in biodistribution and micro-autoradiography studies. Treatment with 2A4 expedited regression of ALκ amyloidomas in mice, likely mediated by the action of macrophages and neutrophils, relative to animals that received a control antibody. These data indicate that the 2A4 mAb might be of interest for potential imaging and immunotherapy in patients with AL amyloidosis.

## Introduction

Immunoglobulin light chain amyloidosis (AL) is a plasma cell dyscrasia wherein monoclonal light chain proteins circulate at high levels and, due to misfolding and seeded aggregation events, accumulate as fibrillar deposits in visceral organs [1]. If untreated, the deposits progress until organ function is compromised and death invariably ensues. About 3000 new cases of AL are diagnosed annually in the USA [2]. The median survival is approximately 3 years, except in patients who present with significant cardiac involvement in which case the prognosis is much worse [3], [4]. A definitive diagnosis is usually made following histological examination of biopsy tissue (usually an abdominal fat aspirate) for the presence of Congo red-birefringent material characteristic of amyloid [5], [6]. Current therapies are primarily directed toward preventing or slowing the production of the amyloidogenic precursor light chain protein, consisting mostly of chemotherapy treatments, with or without stem cell transplantation. More selective therapies employing siRNA and specific antibodies are currently being developed [7]. In certain amyloid-related disorders, notably Alzheimer's disease, passive immunotherapy using amyloid-beta (Aβ) targeted mAbs has been shown to mediate removal of deposits, likely though an opsonization mechanism [8]–[10]. This passive therapeutic approach affords a regulated immunological response, thereby avoiding potential T cell responses associated with active vaccination using fibrils [11]. In like manner, antibodies with specific amyloid binding properties have the potential to bind deposits and promote clearance or mediate neutralization of AL amyloid associated toxicity, possibly reversing or stabilizing the course of the disease [12]–[15].

Another major deficiency in the management of patients with AL amyloidosis is the inability to evaluate directly the whole body disease burden of amyloid and to monitor the response to therapeutic intervention. Although ^123^I-labeled serum amyloid component (SAP) has been used for decades in Europe for detecting visceral amyloid by using planer scintigraphy, it is not approved for use in the U.S., and is not effective in detecting amyloid in some organs [16]–[21]. Recently, the mAb 11-1F4, has been shown capable of imaging visceral amyloid deposits in certain AL patients by using PET/CT [15]. Although effective, both of these agents suffer from an inability to consistently visualize amyloid in the kidneys and heart which importantly lead to the poorest prognoses. Standard imaging methods, including CT and MRI, can detect anatomic defects such as heart wall thickening that are presumed to be due to amyloid; however, these methods are not amyloid specific and are difficult to quantify [22]. For these reasons, other amyloid-reactive reagents including mAbs, may provide additional non-invasive means for detecting amyloid burden *in vivo* by using standard molecular imaging (PET and SPECT) techniques.

Recently we described mAbs for imaging and therapy of AA amyloidosis [23]. This type of amyloid, formed from the sAA precursor protein, generally occurs during periods of chronic inflammation, such as in patients with rheumatoid arthritis or Familial Mediterranean Fever [24]–[26]. During the deposition of AA amyloid, the sAA protein undergoes proteolytic cleavage exposing the C terminal terminal amino acid sequence -Ala-Glu-Asp-Ser- (-AEDS-) (or -His-Glu-Asp-Thr- [-HEDT–] in mice). We have developed antibodies that specifically bind the newly generated cleavage site, but do not recognize the sequence expressed in the full length sAA molecule [23]. There are several examples of mAbs that are poly-reactive and bind multiple types of amyloid fibrils, that differ in the precursor protein from which they are formed [14], [27]–[31], we therefore examined the reactivity of these reagents with other amyloid types.

We report here that *in vitro*, the -HEDT- reactive mAb 2A4 bound with high affinity to AL fibrils as well as human AL amyloid extracts. Furthermore, *in vivo*, it co-localized preferentially in mice with human sc AL amyloid masses as evidenced by SPECT imaging. More importantly, treatment of amyloidoma-bearing mice with mAb 2A4 resulted in an expedited resolution of the amyloid as compared to mice that received a control isotype-matched antibody. The amyloid-specific reactivity parallels with the reaction of mAb 2A4 with sAA as previously demonstrated [23], and provides a basis for the use of this mAb as an agent for imaging and immunotherapy in patients with AL amyloidosis.

## Materials and Methods

### Preparation of the monoclonal antibody and recombinant light chains

The immunogen peptide containing amino acids 71–75 of murine sAA (GHEDT), with the -CG- sequence added for ease of coupling (CGGHEDT), was synthesized by Anaspec Inc. (Fremont, CA) along with an extended peptide that served as a control (-GHEDTMADQE-) for the uncleaved SAA sequence. The immunogen peptide was conjugated to sheep anti-mouse IgG using *N*-[ε-maleimidocaproyloxy]succinimide ester (EMCS) to crosslink the cysteine to the tertiary amines on lysyl residues in the sheep antibody in order to enhance immunogenicity of the peptide. Details of the hybridoma production, screening and culture have been published elsewhere [23]. Briefly, hybridomas from the immunized mice were screened and 3 mAbs selected for specific binding to the immunogen, as compared to a control peptide construct. Of the 3 selected antibodies 2A4 was chosen for further study. The hybridoma was recloned and the product, IgG_2b_, was purified for these studies [23]. MOPC 141 and JH70, both murine IgG_2b_ reagents, were used as a subclass-matched control mAbs. Recombinant (r) Vλ6Wil and Jto proteins were produced and purified as previously described [32].

### Biotinylation of purified antibodies

Purified antibodies were diluted to 1 mg/ml in PBS and biotinylated with a 10-fold molar excess of EZ-Link NHS-PEG4-Biotin, No Weigh Format (ThermoFisher Scientific, Waltham MA), according to the manufacturer's specifications.

### Immunohistochemistry

Six µm-thick sections, cut from formalin-fixed, paraffin-embedded tissues, were subjected to antigen retrieval using the High pH Target Retrieval™ system (Dako, Carpenteria, CA). After a 30-minute incubation at 90°C, followed by 20 minutes at room temperature, the AL amyloid-laden tissues were immunostained with biotinylated 2A4 (3 µg/mL in 0.05% Tween 20/PBS) by overnight incubation at 4°C. The reaction was developed using the Vectastain ABC kit (Vector Labs, Burlingame, CA).

### Surface plasmon resonance

Surface plasmon resonance measurements were made on a BIAcore X instrument. Synthetic recombinant Vλ6WIL fibrils or Vλ6WIL monomer protein were coupled to Fc1 or Fc2, respectively of a CM-5 chip (GE Healthcare) at 200 µg/mL in pH 4.0 sodium acetate buffer using amino chemistry, as described by the supplier. Fibrils were disrupted for 10 sec using a probe sonicator prior to coupling. Dilutions of antibodies or antibodies mixed with peptides in HSB-EP buffer (GE Healthcare) were injected (70 µL) at a flow rate of 20 µL/minutes using a 200 sec delay wash protocol. Raw data were analyzed with BIAcore software for kinetic parameters and were transferred to Excel files for plotting.

### Radiolabelling

The mAbs (100–200 µg) were oxidatively radioiodinated with 2 mCi of reductant-free ^125^I (Perkin Elmer), using 5 µg of chloramine T [33]. After quenching the reaction with 5 µg sodium metabisulfite, the mixture was diluted in PBS containing 5 mg/ml bovine serum albumin (BSA). Unbound isotope and protein aggregates were removed from radiolabeled monomer IgG by size-exclusion liquid chromatography using an Ultrogel AcA34 column (Amersham Pharmacia, Piscataway, NJ). Radiolabeling efficiency was qualitatively assessed by Cyclone phosphor imaging (Packard Instruments, Waltham, MA) of ^125^I-labeled mAb in 10% SDS/PAGE gels, under non-reducing or reducing conditions.

### Small animal SPECT/CT imaging

The biodistribution of the radiolabeled mAbs was determined in groups composed of 3 control or amyloidoma bearing mice (see below). Each animal was injected with ∼6 µg of ^125^I-labeled mAb (∼150 µCi) in the lateral tail vein and, after 48 h, given a 200 µL i.v. dose of vascular contrast agent Fenestra VC™ (Advanced Research Technologies, Montreal, Canada). The vascular contrast agent was added to distinguish blood pool from extra-vascular deposition in the target tissues. Thirty minutes later, the mice were sacrificed by isoflurane overdose and SPECT/CT images acquired.

SPECT data were collected with a microCAT II+SPECT dual modality imaging apparatus equipped with a 1.0 mm-pore diameter pinhole collimator (Siemens Preclinical Solutions, Knoxville, TN), as previously described [23]. Images were reconstructed using an implementation of the expectation maximization-maximum likelihood (EM-ML) algorithm.

After acquisition of SPECT data, high-resolution CT images were obtained with the microCAT II scanner, as previously described [23]. Images were reconstructed in real-time on isotropic 77 µm voxels using an implementation of the Feldkamp back projection algorithm. Datasets were visualized and co-registered manually with a 3-D image analysis software package (Amira, Version 3.1: Mercury Computer Systems, Chelmsford, MA). These studies were carried out in strict accordance with a protocol approved by the University of Tennessee Institutional Animal Care and Use Committee. All procedures were approved by the IACUC and were performed in accordance with the guidelines provided by OLAW and the Guide for the Care and Use of Laboratory Animals.

### Biodistribution

Tissue samples harvested from amyloidoma-bearing and control mice injected with the ^125^I-labeled mAbs were harvested at necropsy after SPECT/CT imaging and were placed into tared vials, weighed, and the radioactivity measured. The primary index values were expressed as % injected dose/g tissue (% ID/g).

### Autoradiography

Six µm-thick sections cut from formalin-fixed, paraffin-embedded blocks of tissue obtained from mice sacrificed 48 h post-injection of ^125^I-labeled mAb were placed on Probond microscopic slides (ThermoFisher), dipped in NTB-2 emulsion (Eastman Kodak, Rochester, NY), stored in the dark, and developed after a 96 h exposure. The sections were counterstained with hematoxylin and eosin, cover-slipped with Permount (ThermoFisher), and examined by light microscopy (Leica DMR epifluorescent microscope). In addition, consecutively-cut sections were stained with Congo red and viewed under cross-polarized illumination. Digital images were captured and evaluated with the Image Pro Plus software package (MediaCybernetics, Bethesda, MD).

### Binding of 2A4 mAb to human AL amyloid extracts

Fibrils (50 µL of 0.83 µM) or amyloid extracts (amounts normalized to the ThT fluorescence) were dried onto Costar EIA/RIA high bond 96 well plates from 50 µL aliquots at 37°C. Wells were blocked for 1 h with 200 µL of 1% BSA in PBS. Primary antibodies with or without inhibitors and secondary antibodies were added in 100 µL of a diluent of 1% BSA and 0.05% tween 20 in PBS. For ELISA assays the secondary antibody was 1/10,000 dilution of HRP goat anti-mouse IgG (Jackson) developed with an ATBS/H_2_O_2_ substrate for 30 minutes before reading on a Victor 3 Wallac microplate reader (Perkin Elmer). Europium based assays (EuLISA) were completed with 1/3000 dilution of biotinylated goat anti-mouse IgG (Sigma) for 1 h followed by 1/1000 dilution of europium/streptavidin (Perkin Elmer) for 1 h and developed with Perkin Elmer enhancement solution before reading on the Wallac system. The use of autopsy-derived human amyloid material in this study was approved by the University of Tennessee Graduate School of Medicine Institutional Review Board (#1007). Materials were obtained following written informed consent.

### Analysis of Kir Bence Jones Protein BJp

Purified and lyophilized Kir Bjp was dissolved at 3.5 mg/ml in PBS plus 0.05% NaN_3_. After brief sonication and centrifugation at 20,000×g for 5 minutes the sample was loaded onto a Hiload 16/60 Superdex 75 prep grade size exclusion chromatography resin (GE Healthcare). One-mL fractions were collected with a 1 mL/minute flow rate. SDS-PAGE was performed on reduced or unreduced selected column fractions using 4–12% bis-tris gels with MES buffer. The fractions were evaluated for protein concentration by measuring the absorbance at 280 nm and used in a competition EuLISA, as described previously.

### Thioflavin T fluorescence measurement of AL extracts

Human AL amyloid extracts were resuspended at 1 mg/mL in PBS. Samples of 10 µL were added to 30 µL of PBS and 10 µL of ThT solution (300 µM stock) and the fluorescence intensity at 490 nm measured using an excitation of 450 nm.

### Immunotherapy procedure

An extract enriched in amyloid deposits by differential centrifugation of human autopsy-derived liver from a patient (Hig) with κ1 amyloid was prepared and stored as a freeze dried powder. The powder was suspended in PBS by treatment with a polytron homogenizer at a concentration of ∼50 mg/250 µL PBS. The resulting slurry was injected subcutaneously between the scapulae of immunodeficient (scid/beige) mice (Charles River). Over the course of 1 week, amyloid deposits formed tumor-like vascularized structures termed amyloidomas [13]. Animals bearing amyloidomas were used for SPECT/CT imaging and for immunotherapy studies. For the immunotherapy, groups of 7 mice were treated on days 7, 10, 13, 15, 17, 20, 22 and 24 post implantation of the amyloid with 100 µg of antibody 2A4 or control JH70 in ∼100 µl PBS, by sc injection on the lower flank of the animal (a site distant from the amyloidoma). Mice were examined by microCT scans on days 7 and 28 to determine amyloidoma size. Mice were sacrificed at day 28 and the amyloidomas were excised, weighed and photographed before fixation in buffered formalin in preparation for immunohistochemistry. Tissue sections were prepared as described above and immunostained by overnight incubation at 4°C using a 1∶9000 dilution of rabbit anti-mouse Iba-1 polyclonal Ab (Wako Pure Chemicals USA, Richmond, VA). Immunohistochemical stains were developed as described above.

## Results

**Figure 1:**
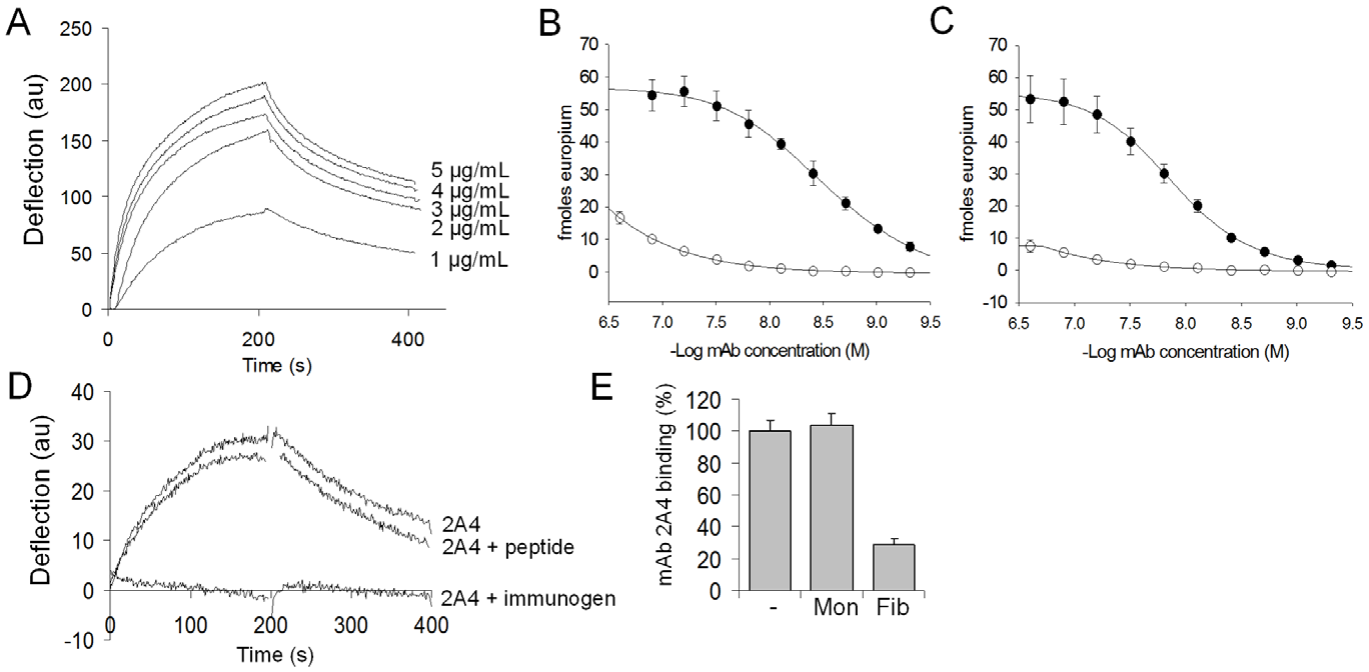
Binding of mAb 2A4 to synthetic AL-derived amyloid fibrils. A) Concentration-dependent binding of mAb 2A4 assessed by SPR analyses. B&C) EuLISA of mAb 2A4 (closed circles) or control mAb JH70 (open circles) concentration dependent binding for rV_λ_6 Wil (B) or Jto (C) synthetic fibrils coated at 0.83 µM. (D) SPR analysis of mAb 2A4 binding to rV_λ_6Wil fibrils with addition of the immunizing peptide (2A4+immunogen), control reagent (2A4+peptide) or alone (2A4). (E) Binding of mAb2A4 to rV_λ_6Wil fibrils coated at 0.83 µM (-) or with addition of Wil fibrils in solution (Fib) or Wil monomer (Mon).

The mAb 2A4 was previously shown to react specifically with the truncated, fibrillar form of sAA [23]. In the course of characterization of the specificity of 2A4, a high-affinity interaction with recombinant AL fibrils was noted. In the present study, we sought to examine further the reactivity of mAb 2A4 with AL fibrils, soluble light chains and human AL amyloid extracts. The 2A4 mAb was shown to bind to fibrils composed of synthetic recombinant Wil and Jto λ6 light chain variable (rV_λ_6) domains by using surface plasmon resonance (SPR) (Fig. 1A) and by EuLISA (Fig. 1b and C). Although the binding kinetics were complex, the K_D_ was estimated to be ∼10 nM in SPR assay (Fig. 1A). The EC_50_ was also determined using EuLISA by titrating the 2A4 mAb or the isotype-matched control reagent, JH70 on surface-adsorbed rV_λ_6 Wil or Jto fibrils (Figs. 1B and C, respectively). The 2A4 mAb bound Wil and Jto rV_λ_6 fibrils with EC_50_ values of 3 and 16 nM, respectively. There was minimal binding of the JH70 mAb even at concentrations >300 nM.

To demonstrate that the reactivity of the 2A4 mAb with fibrils was mediated though the classic antibody-antigen binding site, we performed rV_λ_6 Wil fibril binding studies by using SPR in the presence of the immunogen peptide (-CGGHEDT-) or the read-through control peptide (-GHEDTMADQE-) (Fig. 1D). In the presence of the immunogen peptide the binding of mAb 2A4 to the rV_λ_6 Wil fibrils was completely abrogated, whereas no inhibition was seen in the presence of the control read-through peptide. Similarly, only fibrillar rV_λ_6 Wil was able to effectively compete for the interaction of 2A4 mAb to surface adsorbed rV_λ_6 Wil fibrils in EuLISA assay (Fig. 1E). No competition was seen when monomeric rV_λ_6 Wil was used as the inhibitor in solution even at 20-fold molar excess.

**Figure 2:**
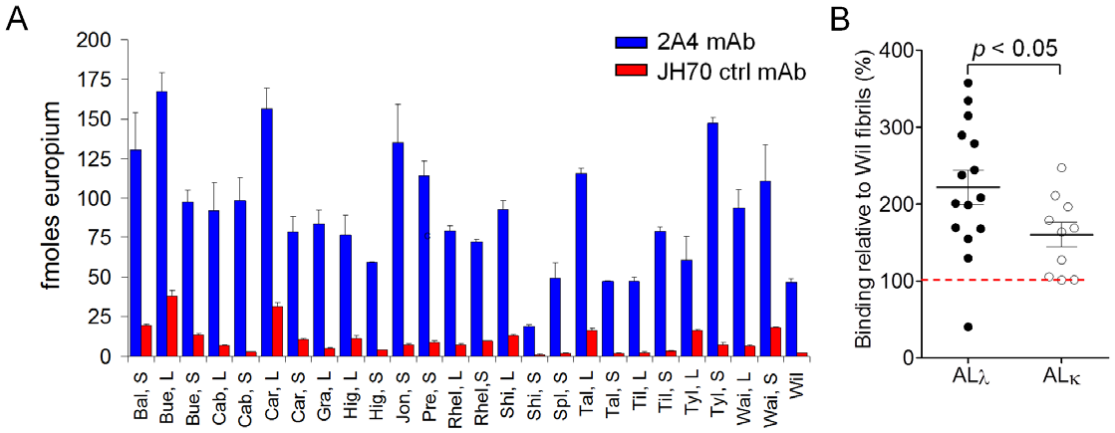
EuLISA of 2A4 (blue) or control JH70 (red) mAb at 50 nM binding to human AL amyloid extracts. (A) Each amyloid extract derived from the liver (L) or spleen (S) was dried onto the well of a 96-well microplate normalized to the ThT signal equivalent to that of 10 µg/mL Hig, L. The far right bars of panel A show binding to synthetic WIL fibrils normalized to the same ThT value. (B) Summary of 2A4 binding to ALκ or ALλ amyloids, normalized to the binding to rV_λ_6WIL synthetic fibrils.

We next examined the reactivity of the 2A4 mAb with human AL amyloid extracts prepared from autopsy-derived material by using EuLISA. The amyloid preparations are composed mainly of fibrils and glycosaminoglycans. Although standard procedures were used to isolate the amyloid, the composition of the extracts has been previously shown to be quite variable (see Table 1). Consequently, the amyloid fibril content per unit weight of each patient-derived extract was estimated by measuring the thioflavin T (ThT) fluorescence (Table 1). To assess the binding of 2A4 with AL extracts, each was coated onto the well of a 96-well microplate at a concentration such that the ThT signal of each was equivalent to that of a chosen standard. i.e., Hig liver (L) amyloid extract at 10 µg/mL (Table 1). The binding of 2A4 (50 nM) to amyloid extracts derived from the liver or spleen of patients with ALκ or ALλ was variable but positive in all cases relative to the binding of the control mAb, JH70 (Fig. 2). With singular exception (Shi, S), the 2A4 mAb bound in greater amounts to all amyloid extracts as compared to the pure fibrillar rV_λ_6 Wil preparation. In addition, the 2A4 mAb bound significantly better to ALλ amyloid as compared to ALκ extracts (Fig. 2B).

**Table 1:** Characteristics of AL amyloid proteins.

Patient (S, spleen; L, liver)	Amyloid subtype	Mean ThT fluorescence (10 ug/mL)	SD	Scaling factor relative to Hig	mAb 2A4 binding relative to Wil (%)
Bal S	λ2	15079.3	948.9	1.6	278.8
Bue L	λ2	11412.7	3707.3	2.1	357.5
Bue S	λ2	16277.3	742.6	1.5	208.3
Cab L	κ4	16542.0	776.4	1.4	196.4
Cab S	κ4	9867.3	129.7	2.4	210.9
Car L	λ2	16146.7	1243.5	1.5	334.6
Car S	λ2	30155.0	954.0	0.8	167.9
Gra L	κ1	16925.3	1260.1	1.4	179.0
Hig L	κ1	23723.7	1527.3	1.0	163.5
Hig S	κ1	13195.7	3331.0	1.8	127.3
Jon S	λ6	23375.7	5262.7	1.0	289.5
Pre S	λ**	38817.0	3925.2	0.6	244.4
RheI L	λ3	23495.7	6431.0	1.0	169.4
RheI S	λ3	12649.3	1297.1	1.9	154.9
Shi L	λ2	41256.7	2663.1	0.6	198.8
Shi S	λ2	75745.3	2580.0	0.3	40.5
Spl S	κ1	24225.3	5393.2	1.0	105.4
Tal L	κ1	17310.7	659.4	1.4	247.2
Tal S	κ1	37507.3	8969.2	0.6	101.6
Til L	κ1	46156.7	6815.9	0.5	101.3
Til S	κ1	46267.3	2326.4	0.5	168.9
Tyl L	λ3	14573.7	2248.4	1.6	129.7
Tyl S	λ3	51178.7	6064.5	0.5	314.9
Wai L	λ2	20947.7	5095.5	1.1	200.8
Wai S	λ2	26827.0	855.0	0.9	237.7

** Subtype is not determined.

**Figure 3:**
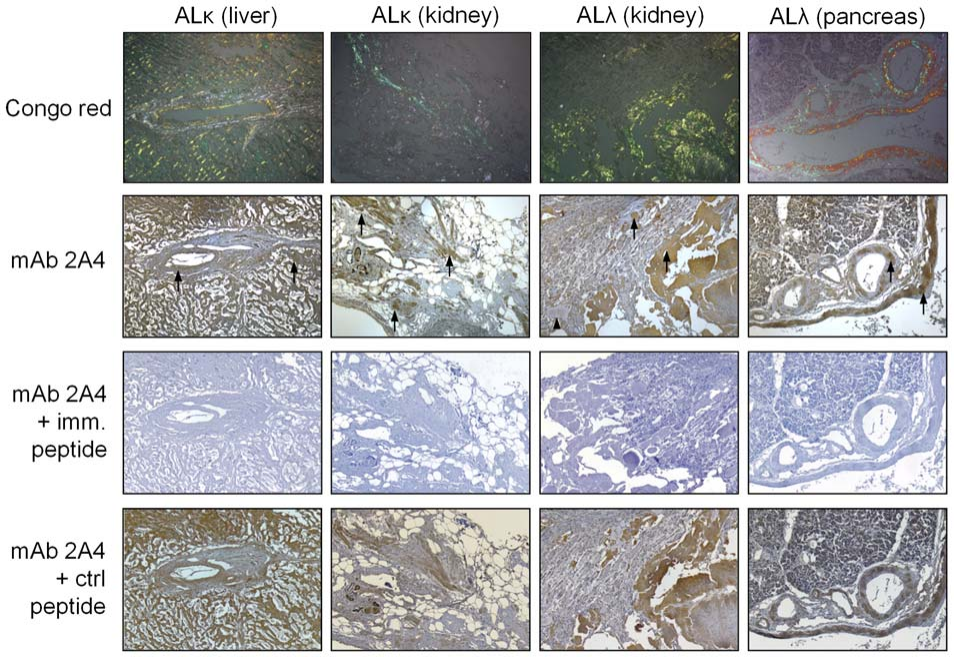
Immunohistochemistry of 2A4 binding to human ALκ and ALλ amyloid in tissue samples. 2A4 mAb was used to immunostain AL amyloid (arrows) in formalin-fixed paraffin-embedded tissues derived from the liver, kidney or pancreas. Top panels show staining with mAb 2A4 alone; middle panels show results with mAb 2A4 adsorbed with immunogen peptide -GHEDT- (imm) and bottom panels mAb 2A4 adsorbed with control peptide -GHETMADQE- (ctrl).

The binding of mAb 2A4 with AL amyloid in tissues was demonstrated using biotinylated mAb for immunostaining of amyloid deposits in formalin-fixed paraffin-embedded tissue sections (Fig. 3). Antibody 2A4 was shown to co-localize with both ALκ and ALλ amyloid deposits in organs such as the liver, kidney and pancreas; however, positive reactivity was only observed in 2/5 ALκ and 5/9 ALλ patient tissue samples. The specific nature of the interaction was evidenced when the reactivity of the mAb was blocked by pre-incubation with the immunogen peptide, but not the read-through control peptide (Fig. 3).

**Figure 4:**
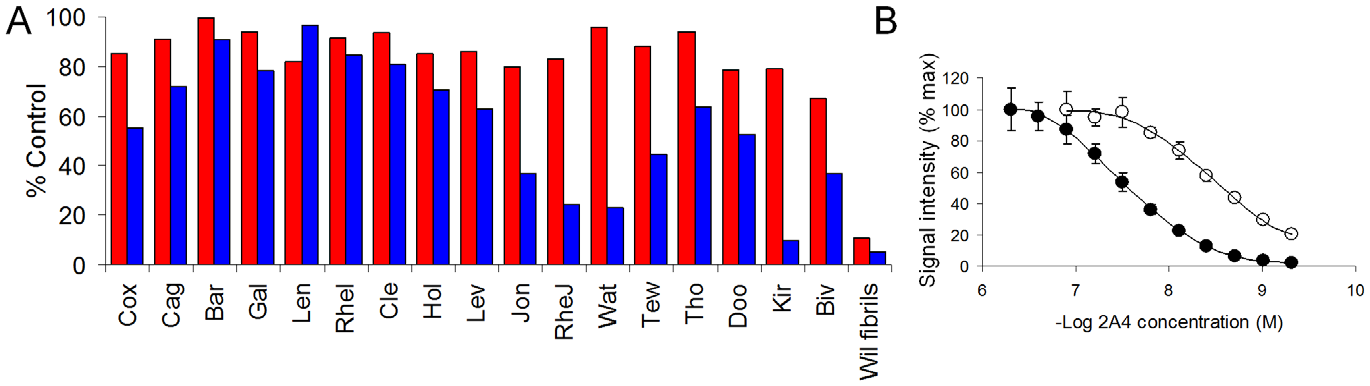
Binding of 2A4 to ALκ Hig amyloid extract and rV_λ_6Wil fibrils in the presence of different Bence Jones proteins. (A) ALκ amyloid extract, 10 µg/mL Hig, L (blue) or 0.83 µM rV_λ_6Wil fibrils (red) were dried onto the wells of a 96-well microplate and the binding of 2A4 evaluated in the presence of BJp pre-incubated with 2A4. Concentrations of 2A4 were 5 nM for wells containing rV_λ_6 Wil (red) and 200 nM for the Hig samples (blue). Competitor BJps, designated on the abscissa by 3 letter code were present at 41.5 µM. The data are expressed as the % of the positive control, i.e., mAb 2A4 in the absence of any BJp. Competition with rV_λ_6 Wil fibrils (far right) served as a positive control. (B) EuLISA titration of 2A4 on Hig, L amyloid extract, 10 µg/mL (closed) and rV_λ_6 Wil fibrils, 0.25 µM (open). The EC_50_ for Wil was ∼3 nM and for Hig, ∼40 nM.

If this mAb is to be useful for imaging deposits in patients, the native circulating light chain should not inhibit binding to amyloid. To examine this, the binding of mAb 2A4 to Hig amyloid extract or synthetic rV_λ_6 Wil fibrils was further assessed in the presence of patient-derived Bence Jones proteins (BJps – urine-derived light chain proteins). Regardless of the presence of most κ or λ BJps as competitors in solution, the 2A4 mAb bound to rV_λ_6 Wil fibrils with <20% loss of reactivity relative to control binding (Fig. 4A). In contrast, addition of certain preparations of BJps, notably Jon (λ6), RheJ (λ3), Wat (κ1), Tew (κ2), and Kir (λ3) inhibited the interaction of 2A4 with Hig amyloid extract by 70–90% (Fig. 4A). Using rV_λ_6Wil fibrils as the competitor blocked >90% of the 2A4 binding to both Hig extract and rV_λ_6Wil fibrils and served as the positive control (Fig. 4A). The apparent ease with which 2A4 binding to Hig could be inhibited by certain BJps (relative to Wil binding) prompted us to determine the EC_50_ for 2A4 on Hig and rV_λ_6Wil fibrils by using EuLISA. The 2A4 mAb bound with greater avidity to rVλ6Wil (∼3 nM) as compared to Hig κ1 (∼40 nM) (Fig. 4B).

**Figure 5:**
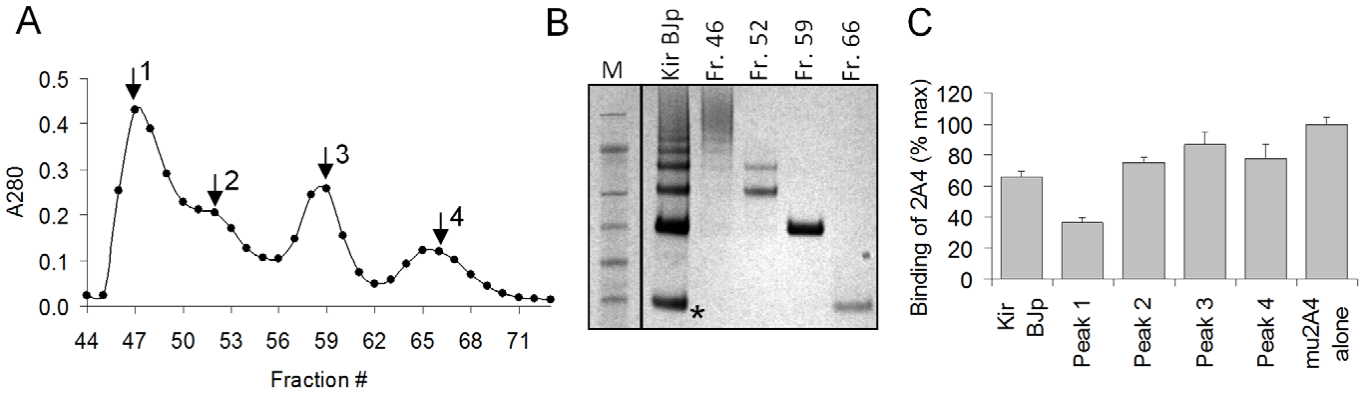
Evaluation of size-fractionated Kir BJp preparation as competitors. (A) Absorbance profile of size fractionated soluble Kir BJp preparation using a Superdex 75 gel filtration column. (B) Fractions, 46, 52, 59 and 66, selected on the basis of the A280 chromatogram trace, were analyzed by SDS-PAGE, and then (C) tested for their ability to inhibit the reaction between 2A4 (200 nM) and Hig L amyloid extract (10 µg/mL) in the standard ELISA. Each fraction was used at the same concentration based on A280 to inhibit binding as described for Fig. 4A.

Certain BJp preparations were effective inhibitors of the binding of 2A4 to Hig amyloid extract (Fig. 4a). We hypothesized that aggregates of light chains in these BJP preparations could expose cryptic epitopes and cause the inhibitory effects. To test this hypothesis, the Kir BJp was rehydrated and fractionated by size-exclusion FPLC. Four distinct peaks were resolved in the eluted protein fractions, ranging in molecular weight from 24 to >200 kDa, (Fig. 5A). The large molecular species (presumably light chain aggregates) were present in the original Kir BJp preparation and were isolated with relatively good purity as assessed by SDS-PAGE (Fig. 5B). Each fraction was tested for its ability to inhibit the binding of 2A4 to Hig amyloid extract as before. The greatest inhibition resulted from the addition of the highest molecular weight aggregates found in fraction 46 (∼70% relative to 2A4 mAb alone). The monomer and dimer Kir BJp caused only ∼20% reduction in the binding to Hig extract, near the background level.

**Figure 6:**
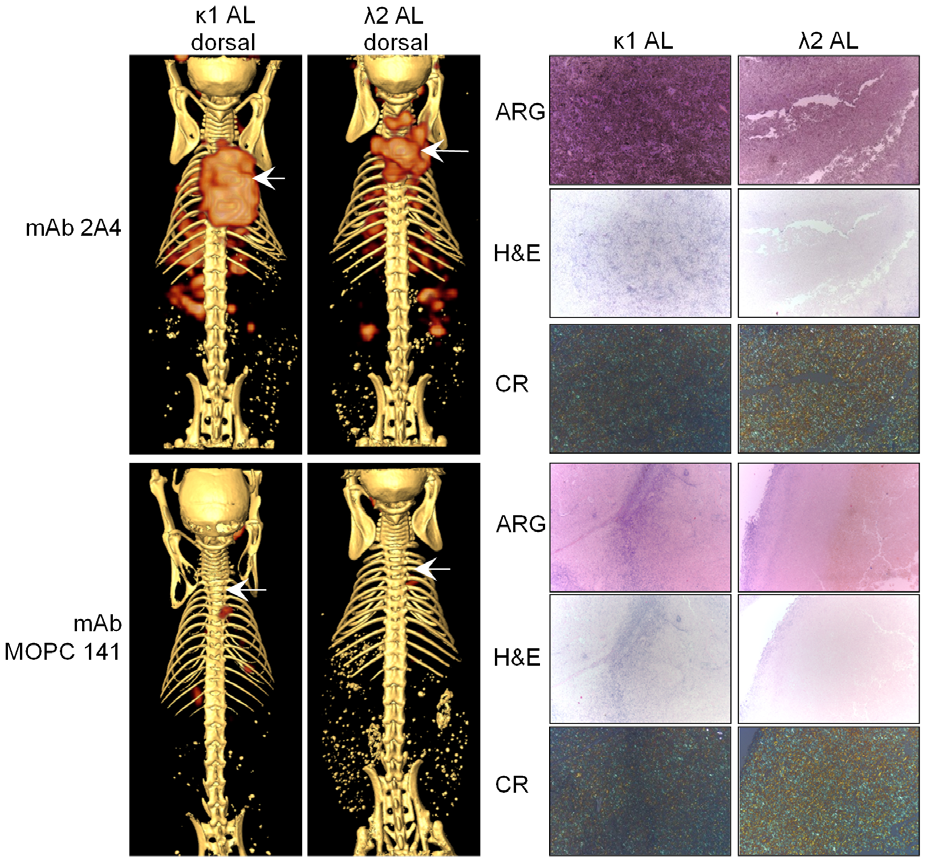
Binding of radioiodinated 2A4 or isotype matched control IgG MOPC 141 to human ALκ and ALλ amyloidomas. Antibodies were radiolabeled with ^125^I and injected iv in the lateral tail vein (∼150 µCi, 25 µg) of mice bearing human AL amyloidomas (50 mg of human κ1 AL [Hig] or λ2 AL [Shi]), at 7 days post-implantation. Mice were imaged post mortem at 48 h post injection using SPECT/CT and the site of the amyloidoma was determined from the CT imaging and is indicated by the arrows (left panels). Tissues including the amyloid mass were harvested, fixed and sectioned for analysis. The right hand panels show autoradiography of the radioiodine tracer (ARG), hematoxylin and eosin staining for tissue morphology (H&E) or staining with Congo red to show amyloid deposits (CR).

After demonstrating reactivity of the 2A4 mAb with synthetic fibrils and human AL amyloid extracts *in vitro* we evaluated this interaction *in vivo*. The 2A4 mAb and the isotype matched control reagent, MOPC141 were radiolabeled and injected iv into mice bearing either Hig (L) κ1 or Shi (L) λ2 sub-cutaneous human AL amyloidomas. Forty-eight hours post-injection the mice were euthanized and the biodistribution of the mAbs assessed by using small animal SPECT/CT imaging and with biodistribution and microautoradiographic analyses. Accumulation of the ^125^I-labeled 2A4 mAb, but not the MOPC-141, in the subcutaneous amyloidoma was evidenced in the SPECT images (Fig. 6). Although both murine monoclonal antibodies were of the same subclass, MOPC 141 appeared to clear from the circulation faster that did mAb 2A4. For this reason we compared tissue∶amyloidoma ratios to assess binding specificity (Tables 2 & 3). Based on the imaging and biodistribution data it appeared that the 2A4 bound in greater quantity to the ALκ amyloidoma as compared to the ALλ mass. This qualitative assessment was confirmed when, after the SPECT/CT procedure, the radioactivity in each tissue was measured (Tables 2 & 3). There was ∼2-fold more ^125^I in the ALκ amyloidoma (10% ID/g) as compared to 5.4%ID/g in the ALλ mass. This translated into correspondingly higher amyloid∶organ ratios in the ALκ amyloid-bearing mice where, in all cases, the ratio was >2. In contrast, only the amyloid∶spleen ratio was >2 in the mice bearing the ALλ amyloidomas (Tables 2 & 3).

**Table 2:** Hig biodistribution and amyloid∶organ ratio.

	Biodistribution (%ID/g)		Biodistribution (%ID/g)		Amyloid∶Organ ratio	Amyloid∶Organ ratio
	2A4		MOPC 141		2A4	MOPC 141
	Mean	SD	Mean	SD		
Amyloidoma	10.0	4.6	2.4	0.4	1.0	1.0
Liver	3.5	0.2	1.9	0.1	2.9	1.3
Spleen	2.6	0.5	1.0	0.1	3.8	2.3
Kidney	4.8	0.5	1.5	0.1	2.1	1.6
Heart	5.0	0.5	1.4	0.3	2.0	1.7

**Table 3:** Shi biodistribution and amyloid∶organ ratio.

	Biodistribution (%ID/g)		Biodistribution (%ID/g)		Amyloid∶Organ ratio	Amyloid∶Organ ratio
	2A4		MOPC 141		2A4	MOPC 141
	Mean	SD	Mean	SD		
Amyloidoma	5.4	2.1	1.4	1.1	1.0	1.0
Liver	3.0	0.6	1.3	0.2	1.8	1.1
Spleen	1.9	0.5	0.8	0.2	2.8	1.8
Kidney	4.1	0.3	1.3	0.1	1.3	1.1
Heart	4.1	0.4	1.4	0.5	1.3	1.0

**Figure 7:**
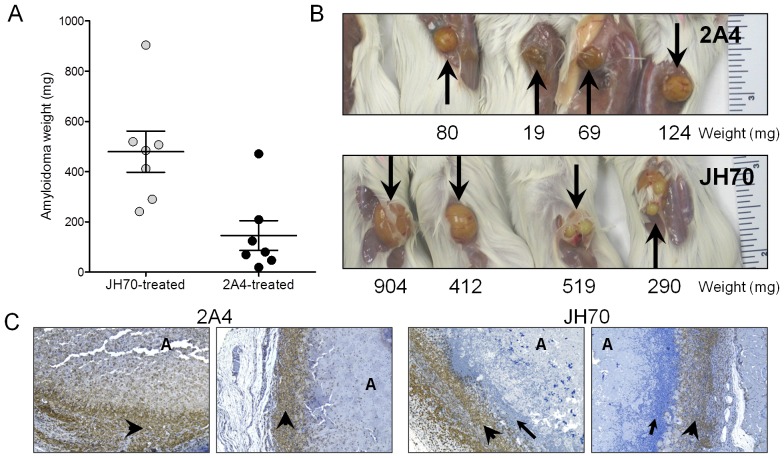
Effect of mAb 2A4 on the resolution of human ALκ amyloidomas in mice. The mAbs 2A4 or JH70 were administered subcutaneously into scid mice bearing human ALκ amyloidomas (50 mg Hig extract), 7 days after injection. Mice were euthanized and the mass of the residual amyloidoma was measured (A) and the amyloidomas photographed (B). Amyloidomas were fixed and processed for paraffin sectioning. Panel C shows immunohistochemical staining for macrophages (anti-Iba-1) in representative sections of amyloidomas from mice treated either with mAbs 2A4 or JH70. Arrowheads designate the positive staining for macrophages; arrows are the band of polymorphonuclear cells, and; “A” designates the major mass of amyloid material.

We had previously used this murine model of human amyloidoma to demonstrate the amyloid reactivity and therapeutic efficacy of the 11-1F4 mAb [12], [13], [34]. We therefore performed similar studies using the 2A4 mAb. Cohorts of mice bearing 50 mg human ALκ (Hig, L) amyloidomas were prepared by injecting precisely equivalent volumes of the stock amyloid slurry subcutaneously on the back. The mice were treated at a site away from the amyloidoma by sc injection of 100 µg 2A4 mAb for 4 wk (2 injections per week). For these experiments, the mAb JH70 served as a control. The amyloidomas, excised post-mortem, were photographed and weighed. The 2A4-treated mice had, on average, significantly smaller masses with a mean weight of 146±150 mg, as compared to the JH70 control mAb-treated animals with amyloid masses averaging 420±260 mg (*p* = 0.03) (Fig. 7A). Gross anatomy confirmed that the size of the amyloid masses excised from 2A4-treated mice were much smaller relative to those from the JH70-treated animals (Fig. 7B). The amyloidomas from 2A4-treated mice were characterized microscopically by the presence of Iba-1-positive macrophages (arrowheads) at the periphery of the mass and infiltrating the amyloid (Fig. 7C). Although the presence of amyloid laden macrophages (with a pseudo-“foamy” appearance) was noted (arrowheads), there was no evidence of multi-nuclear giant cells in the macrophage population. The amyloid lesions isolated from mice that received the JH70 mAb contained, in addition to macrophages located on the periphery, a prominent band of polymorphonuclear cells (arrow) that infiltrated the amyloid, which may be indicative of an IgG-independent neutrophil and macrophage reaction to the foreign amyloid material (Fig. 7C).

## Discussion

Amyloid is a complex extracellular matrix composed of protease-resistant fibrils and a plethora of accessory molecules that accumulate in organs and tissues causing dysfunction and ultimately contribute to significant morbidity and death. To date, most therapeutic approaches for AL and SAA amyloidoses have focused on preventing the accumulation of amyloid by destroying the source of the precursor protein or alleviating the underlying cause of the precursor protein production. To this end, high dose chemotherapy is used to non-specifically eliminate monoclonal plasma cells in patients with AL, and anti-inflammatory agents are employed to halt the production of the acute phase sAA protein. Other approaches include the use of small molecules to block protein-protein interactions [35], [36], orthotopic liver transplantation for patients with hereditary ATTR [37], and more recently, small interfering RNA techniques have been used to specifically target and reduce the production of amyloid precursor proteins [38]–[40].

Although these approaches can elicit therapeutic benefit by reducing the progression of amyloid deposition, there are currently no clinical options capable of actively removing visceral amyloid deposits from affected tissues. However, this goal has been achieved clinically in patients with Alzheimer's disease (AD) [41], [42] and preclinically in experimental murine models of AD and AL amyloidosis by using amyloid-reactive antibodies as part of an immunotherapy protocol [8]–[10], [12]. In each of these cases, the mAbs have been shown to bind to the deposits and then mediate their removal (“amyloidolysis”). Each of these mAbs binds a unique component of the amyloid. The mAb bapineuzumab, which has recently completed phase 3 clinical trials in patients with AD, binds specifically to the *N*-terminal of the Aβ peptide [41], [43]. In contrast, the mAb 11-1F4, which is in Phase I biodistribution studies in man, binds a conformational epitope (a putative type VI β-turn) formed at the N-terminal of human light chain proteins when they are partially denatured or incorporated into the amyloid fibril [14], [15]. Finally, Pepys and Hawkins have described an antibody that reacts with amyloid-associated serum amyloid P component (SAP), following clearance of circulating SAP using the small molecule, *R*-1-[6-[*R*-2-carboxypyrrolidin-1-yl]-6-oxo-hexanol] pyrrolindine-2-carboxylic acid (CPHPC). The anti-SAP mAb, opsonized amyloid and, in mice, was shown to cause rapid resolution of AA amyloid from visceral organs [44].

Although both 2A4 and 11-1F4 react and clear light chain amyloid, they invariably do so by binding to different regions of the light chain fibril. The 2A4 mAb, like 11-1F4, may also provide a novel method for imaging the distribution of amyloid in patients because of its preferential reactivity with an epitope present on AL amyloid fibrils, but not on the circulating light chain precursors. Both antibodies bind to human AL amyloid extracts, which also contain many of the accessory proteins that are present *in vivo*. Notably, the 2A4 antibody did not immunostain all AL amyloid tissue deposits even though it bound to all amyloid extracts *ex vivo*. It is possible that fixation in formalin resulted in masking the epitope in these cases. This could be due to variation in fixation times and storage of sample in paraffin blocks.

Regarding the possible target of mAb 2A4 on light chain fibrils, this reagent was generated using a peptide that contained the Glu-Asp amino acid pairing and we have hypothesized that the reactivity with AL amyloid fibrils involves a similar cryptic structure that is exposed in the fibrillar (aggregated state) of light chain proteins. Based upon the crystal structure of the rVλ6Wil protein, which exhibits the canonical immunoglobulin fold shared by all κ and λ light chain proteins, the Glu81 side chain is solvent accessible but the Asp82 appears buried in the crystallographic “native state” [45]. It is possible that upon mild denaturation or incorporation into amyloid fibrils the Asp82 side chain becomes exposed and a neo-epitope is generated that is then recognized by the 2A4 mAb. This region of the light chain protein has been shown be prone to structural perturbation and unfolding rendering internal sequences accessible to the chaperone protein BiP [46]. Indeed, a peptide based on the light chain residues 72–78 has been shown to prevent the in vitro fibrillogenesis of rVκ4 proteins, implying that this region becomes exposed and may be involved in light chain-light chain interactions in the fibril [47]. The hypothesis that the epitope is revealed in unfolded light chains is further supported by our studies on the Kir Bjp preparation. Our data show that when this BJp was aggregated competing epitopes were present that were essentially absent in the monomeric (presumably correctly folded) form. This epitope hypothesis is presently under investigation.

Even though the precise structure of the epitope on AL amyloid fibrils has yet to be discerned, the 2A4 mAb effectively bound synthetic fibrils and human AL amyloid extracts, even in the presence of a 20-fold molar excess of free BJp. In this study we identified certain BJp preparations that did effectively compete for the amyloid binding of 2A4 to Hig amyloid, but not rV_λ_6Wil fibrils, presumably due to the greater affinity of mAb 2A4 for the latter. We found that this phenomenon likely occurred because certain BJp preparations contained significant aggregates that were responsible for eliciting this effect. It is unknown at this time whether these aggregates, which presumably consist of non-natively folded light chain protein (hence the reactivity with 2A4) are present in patient serum or are an artifact generated during lyophilization and storage of the BJp or induced by its presence in urine [48].

Animal models of AL amyloidosis are limited. The amyloidoma model used in this study, although the best available for this disease, is much different than the disease in patients. Organ site, vascularization and other differences temper the relevance of these results to the human condition. For example, the binding of 2A4 to Hig and Shi liver extracts *in vitro* was essentially equivalent when the extracts were dried on to the microplate and the reactivity with 2A4 assessed by EuLISA. In contrast, ^125^I-2A4 bound to Hig amyloidoma *in vivo* at 2-fold higher amounts as compared to the Shi amyloid mass. There are many factors that can affect the binding of antibody *in vivo* and the dissolution of an amyloidoma. Packing of the fibrils, the presence of accessory molecules associated with the amyloid deposit, and the vascularity of the deposit itself are likely important. Practically speaking, we have previously shown that immunologic dissolution of human ALλ amyloidoma in untreated mice was slower than that for ALκ, which may similarly reflect some of the differences mentioned above.

Despite these deficiencies however, the amyloidoma model can be used to evaluate the targeting and opsonization effects of mAbs in much the same way that subcutaneous tumor xenografts are employed in mice in preclinical oncology studies. In the studies present here, mAb 2A4 was shown to bind the amyloid mass *in vivo* and to accelerate the resolution of the amyloidoma as compared to a control reagent (Fig. 7). Furthermore, immune cell recruitment at the amyloidoma site was characterized by a much different pattern than was seen in control Ab-treated mice.

Preliminary work reported here indicates that 2A4 mAb may represent the latest in a growing armory of potential amyloid-resolving reagents. Although the mouse amyloidoma model has limitations relative to human AL, the ability of mAb 2A4 to opsonize the amyloid deposits and mediate the recruitment of immune cells augur well for its potential as a therapeutic. Radiolabeled mAb 2A4 may also serve as a novel tool for radioimmunoimaging of amyloid *in vivo*. It exhibits remarkable preferential reactivity for AL amyloid in the presence of BJp's. Characterization of a humanized form of 2A4 is in progress.
